# Cellular Profile of Subfornical Organ Insulin Receptors in Mice

**DOI:** 10.3390/biom14101256

**Published:** 2024-10-04

**Authors:** Han-Rae Kim, Jin-Kwon Jeong, Colin N. Young

**Affiliations:** Department of Pharmacology and Physiology, George Washington University School of Medicine and Health Sciences, Washington, DC 20037, USA; hrkim@gwu.edu (H.-R.K.); jinkwon0911@gmail.com (J.-K.J.)

**Keywords:** circumventricular organ, neurons, astrocytes, angiotensin-II, insulin

## Abstract

Brain insulin receptor signaling is strongly implicated in cardiovascular and metabolic physiological regulation. In particular, we recently demonstrated that insulin receptors within the subfornical organ (SFO) play a tonic role in cardiovascular and metabolic regulation in mice. The SFO is a forebrain sensory circumventricular organ that regulates cardiometabolic homeostasis due to its direct exposure to the circulation and thus its ability to sense circulating factors, such as insulin. Previous work has demonstrated broad distribution of insulin receptor-expressing cells throughout the entire SFO, indirectly indicating insulin receptor expression in multiple cell types. Based on this, we sought to determine the cellular phenotypes that express insulin receptors within the SFO by combining immunohistochemistry with genetically modified reporter mouse models. Interestingly, SFO neurons, including both excitatory and inhibitory types, were the dominant cell site for insulin receptor expression, although a weak degree of insulin receptor expression was also detected in astrocytes. Moreover, SFO angiotensin type 1a receptor neurons also expressed insulin receptors. Collectively, these anatomical findings indicate the existence of potentially complex cellular networks within the SFO through which insulin signaling can influence physiology and further point to the SFO as a possible brain site for crosstalk between angiotensin-II and insulin.

## 1. Introduction

Alterations in insulin receptor-mediated signaling are directly associated with a variety of cardiometabolic disorders, including obesity, diabetes, hypertriglyceridemia, and hypertension [1,2,3,4,5,6]. In addition to peripheral insulin action, central nervous system (CNS) insulin receptor signaling is critical in the control of metabolic and cardiovascular homeostasis. For example, CNS insulin receptors have been recognized to play a pivotal role in regulating energy homeostasis, including appetite behavior, glucose regulation, adipose metabolism, and energy expenditure [1,4,7,8,9,10]. Findings from animal models and humans have further demonstrated that brain insulin modulates the cardiovascular system, in part, by influencing sympathetic neural outflow to cardiovascular organs [11,12,13,14,15,16,17,18,19]. However, an in-depth understanding of the integrated cellular and neural networks within the brain that mediate insulin receptor-mediated cardiometabolic regulation remains unclear.

Within the brain, neuronal populations have been highlighted as a major cellular target for insulin action in cardiometabolic processes. For example, global neuronal deletion of insulin receptors in mice results in the development of a metabolic syndrome phenotype under normal chow conditions, demonstrating a tonic regulatory role for neuronal insulin receptors in energy homeostasis [1]. In addition, numerous investigations have further demonstrated that insulin signaling within a variety of select hypothalamic neuronal populations (steroidogenic factor 1, single-minded homolog-1, proopiomelanocortin-producing/agouti-related peptide, melanin concentrating hormone-expressing, etc.) are involved in metabolic regulation [4,6,20,21]. However, it is important to consider that insulin receptors are also present on astrocytes in rodents and humans [22,23], and astrocytic insulin receptor involvement in cardiometabolic regulation has also been demonstrated [9,24,25]. For example, mice with deletion of insulin receptors in astrocytes demonstrate blunted brain glucose uptake [26], along with impaired physiological responses to alterations in glucose availability, such as appropriate changes in appetite behavior [9]. Overall, this existing evidence indicates that neuronal cells as well as non-neuronal populations collectively participate in insulin-receptor-mediated metabolic and cardiovascular homeostasis.

We recently reported that insulin receptors are expressed within the subfornical organ (SFO) and play a tonic cardiometabolic regulatory role in mice [27]. The SFO is a forebrain sensory circumventricular organ that lacks a well-established blood–brain barrier (BBB) [28]. Through dense multidirectional projections to cardiovascular and metabolic nuclei [29,30,31,32], the SFO is intimately involved in cardiometabolic regulation due to its ability to sense circulating peripheral and cerebrospinal fluid factors [33,34]. The cellular and anatomical architecture of the SFO is complex; however, SFO angiotensin-II (Ang-II) type 1a receptor (AT_1a_R) signaling pathways in cardiometabolic homeostasis are well-recognized [29,30,34,35,36,37]. Interestingly, histological findings from our previous work revealed broad insulin receptor distribution throughout the rostral to caudal extent of the SFO [27]. This observation indirectly suggests the possible expression of insulin receptors on multiple SFO cell phenotypes, including AT_1a_R neurons. Therefore, we sought to investigate the anatomical characteristics of insulin receptor expression in the SFO by combining genetically modified reporter animal models and histological analyses to improve our understanding of the SFO cellular architecture that may participate in insulin receptor-associated cardiometabolic homeostasis. Our findings demonstrate that insulin receptors are dominantly distributed in neuronal populations, including both excitatory and inhibitory SFO neurons, although insulin receptor expression in astrocytes was also detected in this brain region. Moreover, the majority of SFO AT_1a_R neurons also expressed insulin receptors, suggesting possible crosstalk between Ang-II and insulin signaling within the SFO to regulate cardiovascular and metabolic homeostasis.

## 2. Materials and Methods

### 2.1. Animals

Bacterial artificial chromosome (BAC) transgenic mice in which an eGFP fluorescent reporter is driven by the murine AT_1a_R promoter [B6.FVB(Cg)-Tg(Agtr1a-EGFP)NZ44Gsat/TmilMmucd, stock #036905-UCD, MMRRC, mmrrc.org] [38,39] or mCherry is directed by the endogenous Gad2 promoter/enhancer element (Gad2-T2a-NLS-mCherry, strain #023140, Jackson Laboratory, Bar Harbor, ME, USA) [40,41] were used to visualize AT_1a_R and gamma-aminobutyric acidergic (GABAergic) inhibitory cells, respectively. Reporter and C57Bl/6J mice (strain #000664, Bar Harbor, ME, USA, Jackson Laboratory) were maintained at the George Washington University animal facility with ad libitum access to a normal chow diet and water under a 12 h light/dark cycle. The Institutional Animal Care and Use Committee at The George Washington University approved all experimental procedures following the standard guidelines of the National Institutes of Health Guide for the Care and Use of Laboratory Animals.

### 2.2. Immunohistochemistry

Standard immunohistochemistry procedures were performed as previously described [42]. Briefly, brains were extracted from mice after transcardial perfusion with phosphate buffered saline (PBS, pH 7.4) followed by 3% paraformaldehyde in PBS. The brains were then post-fixed overnight with 20% sucrose at 4 °C, embedded in optimal cutting temperature compound, and frozen with a dry ice/alcohol slurry. Ten 10 µm thick brain coronal sections, including the SFO, were obtained using a cryostat and stored at −80 °C until use. For immunohistochemistry, the sections were air-dried at room temperature (RT), and then, incubated for 30 min in PBS and 60 min in blocking buffer (3% BSA, 0.3% Triton X-100 in PBS) at RT. The sections were incubated with a primary antibody to the insulin receptor diluted in blocking buffer (insulin receptor β: #bs-0681R-Biotin, 1:500, Bioss Antibodies, Woburn, MA, USA) overnight at 4 °C. After repeated washing (3 × 10 min in PBS with slow agitation), the sections were then incubated with a secondary antibody (goat anti-rabbit IgG with Alexa Fluor-488: #ab150077, 1:1000, Abcam, Cambridge, MA, USA) for 2 h at RT. After PBS washing, the sections were incubated again in blocking buffer for 1 h at RT for double immunohistochemistry. Subsequently, the sections were incubated with primary antibodies for the secondary targets (NeuN: #ABN 90, 1:500, Millipore Sigma, Burlington, MA, USA; GFAP: #AB4674, 1:1000, Abcam; CamK-II: #NBP1-51945, 1:150, Novus Biologicals, Centennial, CO; GFP: #AB13970, 1:1000, Abcam, Cambridge, MA, USA) overnight at 4 °C. Following sequential PBS washing, the sections were incubated with secondary antibodies (goat anti-rabbit IgG with Alexa Fluor-594: #AB150080, 1:1000, Abcam; goat anti-chicken IgY with Alexa Fluor-488: #AB150169, 1:1000, Abcam; goat anti-guinea pig IgG with Alexa Fluor-594: #AB150188, 1:1000, Abcam; donkey anti-goat IgG with Alexa Fluor-594: #AB150132, 1:1000, Abcam) for 2 h at RT. The sections were coverslipped with Vectashield mounting medium (#H1500, Vector Laboratories, Burlingame, CA, USA) and visualized using a BX43F Olympus fluorescent microscope (Olympus, Center Valley, PA, USA) with a DP80 camera. Adobe Photoshop was utilized, for presentation purposes, to linearly modify all images.

### 2.3. Quantification and Statistical Analysis

For all analyses, sections were anatomically matched across animals using a mouse brain atlas (Academic Press, San Diego, CA, USA; SFO: between −0.22 and −0.82 mm from Bregma). Co-localization and quantitative analysis were performed using ImageJ 1.52p software (National Institutes of Health, Bethesda, MD, USA; http://rsbweb.nih.gov/ij/; accessed on 21 August 2019). In brief, after creating a composite file of a brain section using the “Merge Channels” option in ImageJ of insulin receptors, cell type marker, and DAPI taken, the “Channels Tool” was used to count the cell type marker-positive cells referencing the DAPI signal. Subsequently, the cells with insulin receptor staining were counted within these cells in a blinded manner. Data are presented as mean ± SEM.

## 3. Results

### 3.1. SFO Insulin Receptors Are Primarily Expressed on Neurons

**Figure 1:**
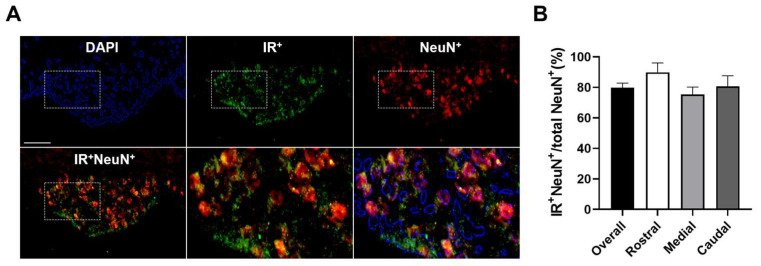
**Expression of insulin receptors on SFO neurons.** (**A**) Double immunohistochemistry and quantification (**B**) for insulin receptors and NeuN, a cellular marker for neuronal populations, in the SFO revealed that a high degree of neurons expressed insulin receptors. DAPI is presented as a nuclei marker. Representative of n = 4. Scale bar = 100 µm.

Our recent investigation demonstrated the distribution of insulin receptors throughout the entire SFO [27]. Moreover, SFO insulin receptor signaling was found to have broad influences on cardiovascular and metabolic physiology [27]. As the SFO utilizes complex cellular and synaptic networks to regulate cardiometabolic homeostasis, it is therefore important to determine cellular phenotypes within the SFO that express the insulin receptor. Based on this, we first investigated neuronal expression of insulin receptors in the SFO using double immunohistochemistry. As shown in Figure 1, NeuN-immunoreactivity (ir), as a molecular marker for neuronal populations, was ubiquitously distributed in the SFO, and 79.8 ± 3.0% of NeuN-ir cells were also labeled with insulin receptor-ir, indicating that broad SFO neuronal populations may express insulin receptors.

**Figure 2:**
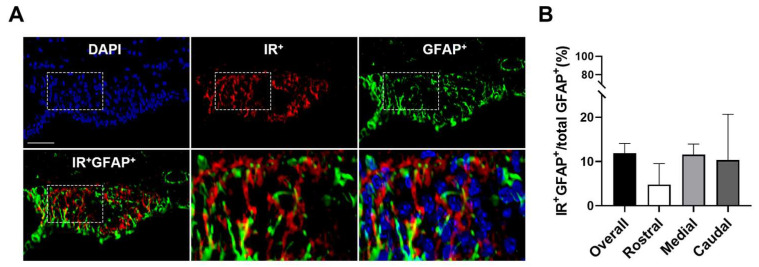
**Expression of insulin receptors on SFO astrocytes.** (**A**) Double immunohistochemistry and quantification (**B**) for insulin receptors and GFAP, a molecular marker for astrocytes, in the SFO revealed moderate astrocytic insulin receptor expression in this brain region. DAPI is presented as a nuclei marker. Representative of n = 3. Scale bar = 50 µm.

We additionally investigated astrocytic insulin receptor expression in the SFO. Consistent with previous reports [43,44], glial fibrillary acidic protein (GFAP) cell body-ir was primarily detected in the rostral to medial SFO regions, following the marginal region to the lateral ventricle, with GFAP-ir fibers projecting toward the core of the SFO. Furthermore, double immunohistochemistry for GFAP and insulin receptors indicated that 11.9% ± 2.2 of SFO GFAP-ir cells were also labeled with insulin receptor-ir (Figure 2). Taken together, these results suggest that SFO neurons are the predominant cell type that express insulin receptors, although a portion of astrocytes demonstrate insulin receptor expression as well.

### 3.2. Insulin Receptors Are Expressed on Inhibitory and Excitatory Neurons in the SFO

**Figure 3:**
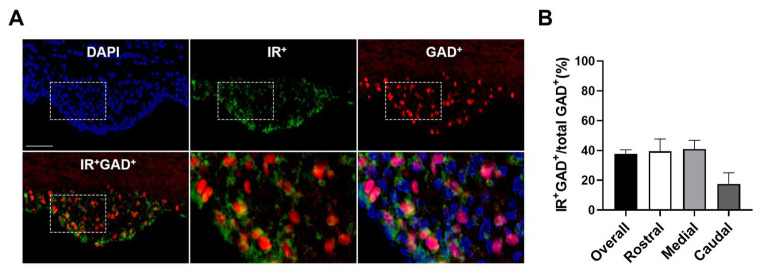
**Expression of insulin receptors on SFO inhibitory neurons.** (**A**) Immunohistochemistry and quantification (**B**) for insulin receptors in GAD-mCherry reporter mice indicate that about one-third of GAD-positive inhibitory cells were also labeled with insulin receptor-ir in the SFO. DAPI is presented as a nuclei marker. Representative of n = 3. Scale bar = 50 µm.

Both excitatory and inhibitory SFO neurons have been recognized to participate in the regulation of cardiometabolic homeostasis [29,37,45,46,47,48,49]. With this in mind, along with the dense neuronal expression of SFO insulin receptors (Figure 1), we further investigated neuronal subtype-specific expression of SFO insulin receptors. Using a GABAergic neuron reporter mouse (GAD-mCherry) combined with insulin receptor immunohistochemistry, we found that 37.6 ± 2.9% of SFO GAD-expressing inhibitory neurons were co-labeled with insulin receptor-ir (Figure 3).

**Figure 4:**
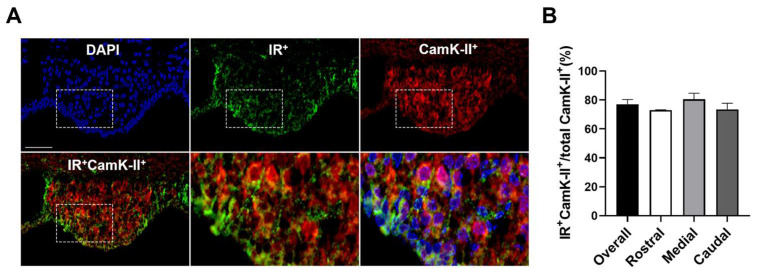
**Expression of insulin receptors on SFO excitatory neurons.** (**A**) Double immunohistochemistry and quantification (**B**) for insulin receptors and CamK-II, a molecular marker for excitatory cells, in the SFO revealed that the majority of excitatory neurons in this brain region were positive with insulin receptor-ir. DAPI is presented as a nuclei marker. Representative of n = 3. Scale bar = 50 µm.

Similarly, double immunohistochemistry for insulin receptors together with Ca^2+^/calmodulin-dependent protein kinase II (CamK-II), a molecular marker for excitatory cells in the SFO [45], indicated that 77.1% ± 3.3 of CamK-II-immunopositive cells in the SFO colocalized with insulin receptor-ir (Figure 4). Therefore, SFO insulin receptors are expressed on both inhibitory and excitatory neurons.

### 3.3. Insulin Receptors Are Expressed on SFO AT_1a_R Neurons

**Figure 5:**
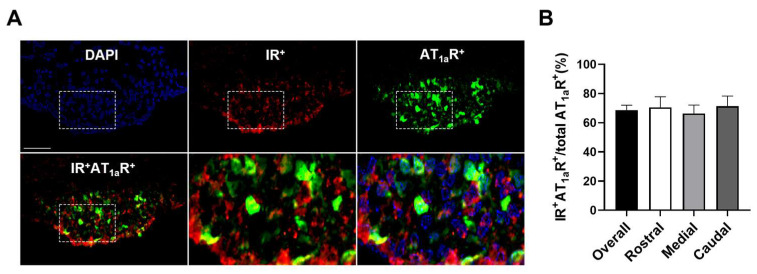
**Expression of insulin receptors on SFO AT_1a_R cells.** (**A**) Double immunohistochemistry and quantification (**B**) for insulin receptor and GFP in the SFO of AT_1a_R-eGFP reporter mice indicate that the majority of AT_1a_R cells, represented by GFP-ir, were colocalized with insulin receptor-ir. DAPI is presented as a nuclei marker. Representative of n = 3. Scale bar = 50 µm.

The SFO has a large density of AT_1a_R-expressing cells, and a central role for angiotensinergic signaling in the SFO in body fluid homeostasis and cardiometabolic regulation has long been recognized [29,30,34,35,36,37]. Importantly, SFO AT_1a_R are solely expressed on neurons [50]. Given that a large degree of SFO neurons also expressed insulin receptors (Figure 1, Figure 3 and Figure 4), we further investigated the possible colocalization of insulin receptors with AT_1a_R in SFO neurons. By combining an AT_1a_R-eGFP reporter mouse line with insulin receptor immunohistochemistry, 68.5% ± 3.5 of SFO AT_1a_R neurons were found to be positive for insulin receptor-ir (Figure 5).

## 4. Discussion

**Figure 6:**
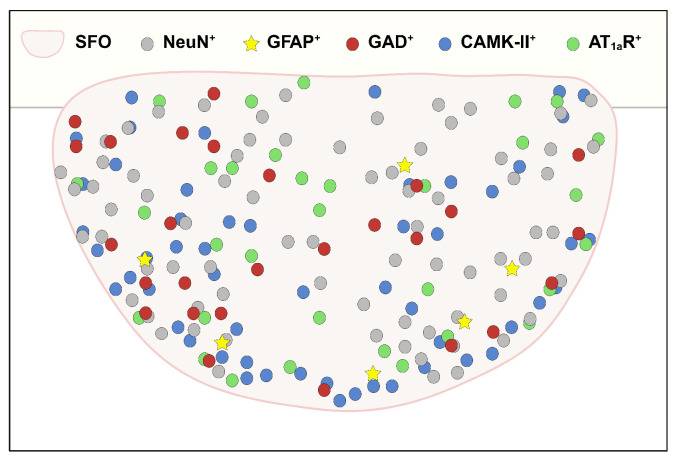
**Schematic illustration showing the regional distribution of NeuN^+^, GFAP^+^, GAD^+^, CamK-II^+^, and AT_1a_R^+^ insulin receptor-positive cells in the SFO.** Note that GAD^+^, CamK-II^+^ and AT_1a_R^+^ cells will also express NeuN and therefore there may be overlap among the illustrated different neuronal populations. Image created with www.biorender.com; accessed on 27 September 2024.

Accumulating evidence strongly supports central insulin receptor signaling in metabolic and cardiovascular physiology [1,4,7,8,9,10,18,19]. In this regard, hypothalamic insulin receptors have been the primary area of focus given the importance of this CNS region in the regulation of neuroendocrine and autonomic output [4,6,20,21]. However, insulin receptors are broadly distributed within the CNS, and multiple studies have also implicated non-hypothalamic insulin receptors in cardiometabolic homeostasis. For example, insulin receptors in brain stem regions, including the ventral tegmental area and substantia nigra, regulate energy homeostasis through modification of catecholaminergic cellular activity [10,51,52]. Independently, insulin receptors in the nucleus tractus solitarius have been shown to play a critical role in physiological as well as pathophysiological cardiovascular regulation [53,54,55]. In line with these results, our recent investigation highlighted the SFO as a forebrain target for insulin in cardiometabolic control [27]. Building upon this, here, we highlight SFO cell phenotype-specific insulin receptor expression (Table 1 and Figure 6), which may lend insight into the complexity of CNS insulin cardiometabolic regulation.

Our anatomical analysis revealed that SFO neuronal cells, including both excitatory and inhibitory neurons, are the dominant cell phenotype that express insulin receptors. In line with this observation, the size and shape of cells with insulin receptor-ir were large and donut shaped, which are the common characteristics of the neuronal population. On the other hand, insulin receptor-ir was also detected in GFAP-ir astrocytes in the SFO as well, although the degree of SFO astrocytic insulin receptor expression was low, relative to neurons. Importantly, multiple cell phenotypes within the SFO have been known to participate in the regulation of cardiometabolic homeostasis. For example, the SFO provides excitatory as well as inhibitory inputs to the hypothalamus to regulate thirst, fluid balance, and cardiometabolic homeostasis [29,37,45,46,47,48]. In addition to inhibitory projecting neurons, involvement of SFO interneurons in cardiometabolic homeostasis, by modulating the activity of SFO projecting neurons, has also been suggested [37,49]. Moreover, SFO astrocytes also have the established molecular machinery to sense circulating fluid information, and subsequently play a role in regulating SFO excitatory and inhibitory neuronal activity [56,57]. Therefore, insulin signaling within a variety of cell phenotypes, including neuronal and non-neuronal populations, within the SFO may participate in cardiometabolic regulation. Moreover, crosstalk between the various insulin receptor-expressing cells in the SFO may occur. For example, excitatory neuronal insulin action may be opposed by inhibitory neuronal insulin action. Although speculative, such interactions warrant further investigation.

Ang-II signaling, as a final product of the renin–angiotensin system, is known to play a central role in regulating cardiometabolic physiology, and impairments in Ang-II signaling within the CNS have been directly implicated in the development of pathophysiological disorders, such as hypertension [58,59,60,61]. Within the CNS, the SFO is well characterized as a direct site of action for circulating Ang-II, with a dense distribution of Ang-II-specific AT_1a_R-expressing cells. Extra-hypothalamic forebrain SFO AT_1a_R provide excitatory inputs to downstream hypothalamic and brainstem nuclei to regulate neuroendocrine outputs, autonomic nervous system outflow, and blood pressure [29,30,37]. Importantly, accumulating investigations in both humans and animal models indicate an interaction between Ang-II and insulin signaling in cardiometabolic regulation [62,63,64,65,66,67]. For example, pharmacological blockade of AT_1a_R attenuated hyperinsulinemia-induced hypertension [62]. On the other hand, chronic low-dose treatment of Ang-II resulted in the development of hypertension that was associated with AT_1a_R-dependent enhanced insulin signaling [65,66]. However, these studies were limited to the periphery, and no information for the centrally mediated crosstalk between Ang-II and insulin in cardiometabolic physiology is available. Our results provide clear anatomical evidence of the co-presence of insulin receptors and AT_1a_R within the same SFO neurons in mice and suggest that the SFO could be the central target for functional interactions between Ang-II and insulin in cardiometabolic regulation. In support of this, multiple studies have demonstrated an anatomical and functional interaction of SFO AT_1a_R with other cardiometabolic signaling molecules. For incidence, recent transcriptome analysis revealed the presence of leptin receptors in the SFO, and leptin-dependent thermogenesis and body weight change in mice were mediated, in part, by SFO AT_1a_R [34,68]. Inflammatory signaling molecules, such as tumor necrosis factor alpha and interleukin-1 beta, have also been suggested to interact with SFO AT_1a_R and synergistically activate SFO-hypothalamic circuits to elevate sympathetic nerve activity and blood pressure [69,70,71,72,73]. Therefore, SFO AT_1a_R mechanisms may not only work independently to influence central cardiometabolic outputs by sensing circulating Ang-II but also by synergistically (and/or opposing) the actions of a broad range of plasma cardiovascular and metabolic metabolites/hormones, presumably including insulin.

## 5. Conclusions

The presence of insulin receptors in the SFO has long been suggested through multi-directional approaches [33,74,75,76,77,78,79]. However, our recent report, for the first time, demonstrated the in vivo function of SFO insulin receptors in the tonic regulation of cardiovascular and metabolic physiology in mice [27]. Here, we provide further anatomical evidence for insulin receptor-associated cellular and neural architecture within the SFO. Insulin receptors are expressed in multiple cell types within the SFO, all of which have been known to participate in cardiometabolic regulation. Therefore, functional dissection of individual cell phenotype-dependent insulin receptor signaling in the SFO is necessary in the future to understand integrated SFO insulin receptor-mediated physiology. Notably, we also demonstrated the expression of insulin receptors within SFO AT_1a_R cells. As mentioned above, the functional interaction of insulin and Ang-II in the periphery in cardiometabolic regulation has been reported. However, both Ang-II and insulin have been known to affect cardiometabolic physiology, in part through CNS mechanisms. In this regard, our results further suggest the SFO as a potential central site where crosstalk between Ang-II and insulin occurs.

## Figures and Tables

**Table 1:** **Summary of colocalization of SFO insulin receptor in NeuN^+^, GFAP^+^, GAD^+^, CamK-II^+^ and AT_1a_R^+^ cells**.

	Insulin Receptor Colocalization (%)			
	Entire SFO	Rostral SFO	Medial SFO	Caudal SFO
**NeuN+**	79.8 ± 3.0	89.8 ± 6.2	75.3 ± 4.9	80.6 ± 7.0
**GFAP^+^**	11.9 ± 2.2	4.8 ± 4.8	11.5 ± 2.4	10.3 ± 10.3
**GAD^+^**	37.6 ± 2.9	39.4 ± 8.2	40.9 ± 5.9	17.5 ± 7.5
**CamK-II^+^**	77.1 ± 3.3	73.0 ± 0.3	80.4 ± 4.3	73.5 ± 4.3
**AT_1a_R^+^**	68.5 ± 3.5	70.4 ± 7.4	66.3 ± 5.8	71.3 ± 7.0

Values are presented as mean ± SEM.

## Data Availability

All the data necessary to support the conclusions of this study are contained within the article.
